# Enhancing emotional intelligence in medical education: a systematic review of interventions

**DOI:** 10.3389/fmed.2025.1587090

**Published:** 2025-07-28

**Authors:** Sabyasachi Maity, Samantha Michelle De Filippis, Alexander Aldanese, Melissa A. McCulloch, Alexis P. Sandor, Jan E. Perez Cajigas, Yiorgos Antoniadis, Te-keila D. T. Rochester, Lauren Elizabeth Carter, Alexander M. Preisig, Julia Ali Kobeissi, Narendra Nayak, Jaime E. Mendoza, Samal Nauhria

**Affiliations:** ^1^Department of Cellular & Integrative Physiology, Long School of Medicine, UT Health San Antonio, San Antonio, TX, United States; ^2^Department of Medicine, University of Medicine and Health Sciences, Basseterre, Saint Kitts and Nevis; ^3^Department of Medicine, St. George’s University School of Medicine, True Blue, Grenada; ^4^Department of Medicine, Ross University School of Medicine, Bridgetown, Barbados; ^5^Department of Microbiology, St Matthew’s University, Georgetown, Cayman Islands; ^6^PrimeWest Consortium, San Dimas Community Hospital, Graduate Medical Education, San Dimas, CA, United States; ^7^Civil Service College, Cayman Islands Government, Georgetown, Cayman Islands

**Keywords:** emotional intelligence, medical students, medical education, empathy, self-awareness, self-management, social awareness, relationship management

## Abstract

**Introduction:**

Emotional intelligence (EI) is a crucial competency for medical professionals, facilitating effective interpersonal relationships between physicians and patients. The ability to evaluate, regulate, and apply emotional understanding plays a significant role in fostering empathy, communication, and stress management. This systematic review aimed to determine the impact of various interventions on medical students’ EI development, academic performance, and overall patient care.

**Methods:**

A comprehensive literature search was conducted for studies published from 2021 until 2024, inclusion criteria focused on studies on medical students, employed validated EI assessment tools, and utilized appropriate research designs.

**Results:**

44 articles met the inclusion criteria. The Joanna Briggs Institute (JBI) Critical Appraisal Checklist was applied to assess the quality of included studies. Although a meta-analysis was initially planned, substantial heterogeneity across the studies limited the pooling of quantitative data. Using an inductive coding approach, eight major themes were identified: Narrative and Storytelling Interventions, Reflective Practices and Writing, Communication Skills Training, Emotional Intelligence Enhancement, Experiential Learning and Patient Exposure, Stress, Burnout, and Coping Interventions, Assessment Tools and Structural Interventions, and Personalized Interventions and Diversity Considerations. These themes were subsequently mapped onto Daniel Goleman’s model of Emotional Intelligence to provide a structured theoretical framework.

**Discussion:**

The findings of this review highlight that various interventions hold promise in enhancing EI among medical students, leading to improvements in personal well-being, communication skills, and professional development. The most effective approach appears to be a multifaceted, longitudinal integration of EI-focused strategies throughout medical training, incorporating repeated practice, guided reflection, faculty mentorship, and structured debriefing. The broader implications extend to improved doctor-patient relationships, reduced burnout, and enhanced clinical decision-making, ultimately contributing to higher patient satisfaction and more compassionate healthcare delivery. Future research should focus on refining intervention methodologies and assessing their long-term impact on medical education and practice.

## Introduction

Emotional intelligence (EI) is a psychological construct first defined as the ability to evaluate and regulate emotions in oneself and in others to achieve positive outcomes (1). Over time, this definition has been expanded to encompass a multidimensional concept involving the use of emotional knowledge to reason accurately with both personal and interpersonal emotions. Understanding EI and its utility has proven to be complex, consisting of much more than a single attribute or skill. Various models have been developed to conceptualize EI, typically categorizing it as a personal trait, an ability, or a combination of both (2).

Several tools are available to evaluate EI in different populations. One longstanding measure is the Levels of Emotional Awareness Scale (LEAS), developed over 30 years ago to assess emotional awareness (3). Specifically targeting healthcare professionals, the Jefferson Scale of Empathy (JSE) has been widely used in research to assess empathy within medical contexts (4).

EI has garnered significant attention due to its association with positive personal, professional, and academic outcomes (5). Recent scholarship increasingly posits EI as a foundational competency within medical education, with significant implications for both clinical performance and professional development. EI encompasses a range of abilities including self-awareness, emotional regulation, empathy, and effective interpersonal communication; skills that are essential not only for managing physician–patient relationships but also for thriving in complex, team-based healthcare environments (6). In the United States, the Accreditation Council for Graduate Medical Education (ACGME) outlines six core competencies: Patient Care, Medical Knowledge, Interpersonal and Communication Skills, Professionalism, Practice-Based Learning and Improvement, and Systems-Based Practice, several of which are directly supported by EI capacities. Specifically, the ability to receive feedback constructively, manage conflict, and demonstrate empathy under pressure are underpinned by EI (7, 8).

This emphasis on EI is echoed in other global medical competency frameworks. The CanMEDS Physician Competency Framework (Royal College of Physicians and Surgeons of Canada) defines roles such as *Communicator*, *Collaborator*, *Leader*, *Health Advocate*, *Scholar*, and *Professional*, all of which presuppose emotionally intelligent behaviors such as compassion, cultural sensitivity, and resilience (9). Some scholars have even advocated for expanding CanMEDS to include a formal “Doctor as Person” role to reflect the importance of self-care and emotional maturity in medical practice (10). Likewise, in the United Kingdom, the GMC Outcomes for Graduates identify essential capabilities including the ability to work effectively within teams, respond to uncertainty, maintain personal well-being, and engage in reflective practice. These outcomes are deeply interwoven with EI, suggesting that emotionally intelligent graduates are better equipped to deliver person-centered, safe, and collaborative care (11).

Empirical evidence further reinforces the value of EI in clinical contexts. Studies have shown that physicians with higher EI are more likely to foster therapeutic alliances, reduce medical errors, manage stress effectively, and prevent burnout (12). These benefits extend beyond individual performance to impact patient outcomes and healthcare system efficiency. A multi-level meta-analysis shows that EI is significantly associated with academic success in MD programs, the strength of this relationship remains modest. Nevertheless, the integration of EI-focused skills into medical curricula and professional development remains valuable for fostering well-rounded, resilient physicians (13).

Given the alignment of EI with national and international medical education standards, the integration of structured, EI-focused interventions throughout undergraduate and postgraduate training is not merely beneficial but essential. Such integration ensures the development of reflective, competent, and emotionally resilient physicians capable of navigating the evolving demands of 21st-century healthcare. However, a major challenge lies in the lack of a unified approach to improving EI in this population (4).

The theoretical foundation for integrating EI in medical education is rooted in multiple frameworks. Various models have been proposed to measure EI in this context. Salovey and Mayer’s model conceptualizes EI as a form of information processing (2). Social Learning Theory suggests that emotional skills can be developed through observation and practice, underscoring the importance of experiential learning. The Bar-On model offers a comprehensive perspective on emotional–social intelligence, emphasizing interrelated emotional and social competencies that influence behavior and performance (14). The Trait Emotional Intelligence Model conceptualizes EI as a set of emotional self-perceptions assessed through self-report questionnaires (15).

For this review, we adopt Daniel Goleman’s EI model as the primary theoretical framework, as it provides a structured approach to addressing the unique challenges medical students face (16). This model emphasizes four key domains: self-awareness, self-management, social awareness, and relationship management. These domains align closely with the demands of medical education and practice (17, 18). Self-awareness, which entails recognizing one’s emotions, strengths, and limitations, is fundamental for managing stress and maintaining mental well-being in rigorous academic and clinical settings. Self-management, which includes emotional self-control, adaptability, and initiative, equips students to navigate high-pressure environments such as clinical rotations while maintaining professionalism (19).

Goleman’s model integrates both personality traits and behavioral competencies, making it highly applicable to professional domains such as medicine (20). His framework extends beyond foundational EI components to include stress management and interpersonal effectiveness, both of which are crucial for medical training (21). Research has demonstrated that higher EI levels correlate with lower burnout rates and greater job satisfaction among healthcare professionals. By integrating EI skill development early in medical training, institutions can better prepare students for the emotional demands of their careers, potentially reducing burnout and improving patient care outcomes.

Previous systematic reviews have highlighted significant variability in how EI interventions are designed, delivered, and assessed (2). Some interventions focus on developing specific EI components, such as empathy or self-regulation, while others evaluate the broader impact of EI training on academic performance and clinical competence. However, inconsistencies remain in the reported effectiveness of these interventions, necessitating a more comprehensive synthesis to determine which approaches yield the most meaningful outcomes.

This systematic review employs Goleman’s model as a framework for conducting a thematic analysis of EI interventions in medical education. Specifically, we aim to: (1) identify various EI-focused interventions, (2) assess their effectiveness in enhancing EI among medical students, (3) compare the impact of different intervention types (e.g., classroom-based versus experiential learning) on EI-related outcomes, and (4) uncover key themes and emerging patterns in the relationship between EI and critical aspects of medical education, such as student performance, well-being, and patient care. By providing a structured thematic synthesis, this review seeks to inform curriculum development and improve the emotional competencies of future healthcare professionals.

## Methods

The study protocol was registered in the International Prospective Register of Systematic Reviews (PROSPERO) under the Center for Reviews and Dissemination at the University of York (CRD42024580108) before the project commenced.

### Eligibility criteria

Following an initial screening of titles and abstracts, full-text articles of selected studies were examined to ensure they met the inclusion criteria. Studies were included if they met the following criteria: (1) the population consisted exclusively of undergraduate medical students enrolled in MD or MBBS programs; (2) the intervention focused primarily on developing or enhancing emotional intelligence (EI) or its related domains such as empathy, emotional regulation, or communication; (3) the study reported measurable outcomes (quantitative, qualitative, or mixed-methods) evaluating the effectiveness of the EI-focused intervention; (4) the intervention outcomes were assessed using validated tools such as the Jefferson Scale of Physician Empathy (JSE-S), Jefferson Scale of Patient Perceptions of Physician Empathy (JPSE), Interpersonal Reactivity Index (IRI), or similar psychometric instruments; (5) the study employed a recognized empirical design, including randomized controlled trials (RCTs), quasi-experimental designs, cohort studies, or pre-post intervention studies; and (6) the article was published in a peer-reviewed journal, in English, from 2021 onward.

Studies were excluded if they: (1) focused on populations outside undergraduate medical education (e.g., nursing students, residents, or non-healthcare students); (2) did not involve an explicit intervention targeting EI or related domains; (3) failed to report original, measurable outcomes post-intervention; or (4) were categorized as literature reviews, editorials, commentaries, conference abstracts, or opinion pieces without primary data.

#### Search strategy

A comprehensive literature search was conducted across multiple electronic databases, including PubMed, CINAHL, Scopus, ERIC, PsycINFO, WOS, and ScienceDirect. The search covered studies published from 2021 onward, restricted to English-language articles. A combination of MeSH terms and free-text keywords such as “emotional intelligence,” “medical education,” “undergraduate,” “interventions,” “training,” “empathy,” and “communication skills” was employed. Boolean operators (“AND,” “OR”) were applied to refine search results, ensuring both sensitivity and specificity. The search process adhered to the Preferred Reporting Items for Systematic Reviews and Meta-Analyses (PRISMA) guidelines (22).

The literature search was limited to studies published from January 2021 onward to capture the most contemporary interventions and trends in emotional intelligence training following the COVID-19 pandemic. This period marked a paradigm shift in medical education emphasizing resilience, well-being, and adaptability which significantly influenced the design and relevance of EI programs. While acknowledging that earlier studies have foundational value, our focus was to synthesize current evidence aligned with the evolving needs and modalities of medical education in the post-pandemic context.

#### Study selection

**Figure 1:**
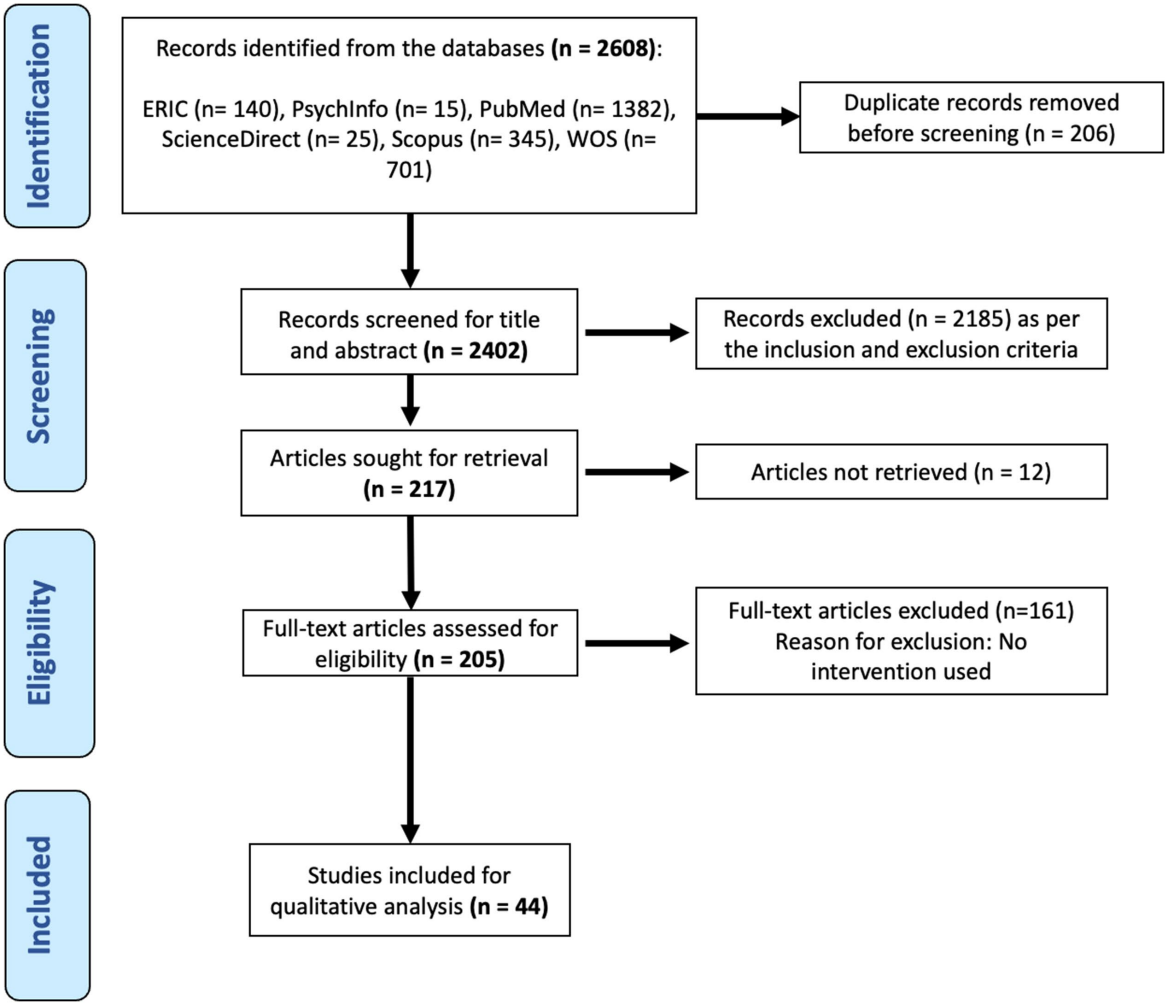
PRISMA protocol of literature search process.

Two independent reviewers screened the titles and abstracts of all retrieved records to identify potentially eligible studies. Full-text articles were subsequently assessed for final inclusion. Any disagreements between reviewers were resolved through discussion or by consulting a third reviewer. The selection process was systematically documented using a PRISMA flow diagram (Figure 1), detailing the number of records identified, screened, and included in the final analysis.

#### Data extraction

Data from 44 included studies (Table 1) were extracted and compiled into an Excel spreadsheet. Extracted variables included “Author,” “Year,” and “Outcome/Conclusion(s)” reported in each study. These data served as the foundation for subsequent analysis.

**Table 1:** Summary of all the articles included in systematic review.

Authors	Country	Aim of study	Study design	Sample size	EI Model used	Outcome/conclusions
Baseer, et al. 2024 (71)	Pakistan	Study the effectiveness of empathy portfolios in developing Professional Identity Formation (PIF) among medical students.	Experimental, Randomized (parallel group)	120, 3rd year medical students	JSPE-5 and PIQ	Empathy portfolios had a positive correlation with PIF but were ineffective as a short-term intervention.
Mahmoudi, et al. 2024 (46)	Iran	Evaluate and compare the impact of story reading on the level of empathy in medical students at Mazandaran University of Medical Sciences in Iran.	Quasi-experimental (controlled trial without randomization)	46 (27 female, 19 male) with an average age of 19.5 years	IRI	Story reading effectively increased medical students’ empathy, with stable effects in follow-up.
Arumugam et al., 2024 (53)	India	Enhance the communication skills of undergraduate medical students, specifically in delivering bad news to patients.	Experimental, Randomized (parallel group)	100 medical students		The EMBRACE module significantly improved students’ ability to deliver bad news using SPIKES protocol.
Kanagasabai et al., 2024 (42)	New Zealand	Investigate the acceptability and efficacy of a patient storytelling intervention (Live and recorded) on empathy levels of medical students.	Experimental, Randomized (parallel group)	22 medical students	JSE-S	Storytelling interventions enhanced medical students’ empathy and understanding of patient experiences.
Rhines et al., 2024 (57)	USA	Evaluate the efficacy and feasibility of a Cognitive Fitness Training (CFT) program on improving emotional intelligence (EI) and stress management.	Experimental, Randomized (parallel group)	80 3rd year medical students, Training Group 42, Control Group 38	Cognitive Fitness Assessment Score	CFT improved EI and cognitive fitness without affecting academic performance, suggesting feasibility for integration.
Kadam et al., 2024 (41)	India	Determine the impact of reflective writing exercises on the emotional intelligence (EI) of medical students.	Observational analytic, Cohort	120 medical students	Bar-on Emotional Quotient Inventory	Reflective writing improved EI, particularly in self-awareness and empathy, and is recommended for curricula.
Britz et al., 2024 (31)	Germany	Compare the impact of simulated patients (SP) vs. real patients (RP) communication training on medical students’ empathy.	Quasi-experimental (controlled trial without randomization)	146 medical students (38.4% male)	JSPE and IRI	SP-group students showed higher external empathy ratings than RP-group students, suggesting SP use early.
Neeley et al., 2024 (45)	Canada	Support student empathy throughout the clinical years of medical school by implementing an empathy-focused intervention.	Quasi-experimental (controlled trial without randomization)	53 medical students participated, with 34 in the curriculum group, 19 in the control	The Toronto Empathy Questionnaire	A brief arts curriculum improved self-reported empathy in students with initially low empathy scores.
Hashim et al., 2024 (40)	Canada	Study reflective writing as a means to nurture empathy among medical students.	Quasi-experimental (controlled trial without randomization)	73 medical students	The Toronto Empathy Questionnaire	Reflective writing did not raise empathy scores but enhanced perspective-taking and compassion.
Blalock et al., 2024 (32)	USA	Address the decline of empathy in third-year medical students by using the skill of noticing as an intervention.	Descriptive, Qualitative	170 third-year medical students	SENT	The study’s outcomes and conclusions centered on the effectiveness of the Social Emotional Noticing Tool (SENT) in enhancing the empathetic practice o.
Khorasani et al., 2023 (56)	Iran	Investigate the effectiveness of an educational intervention based on EI on the level of academic stress components among Iranian medical students.	Quasi-experimental (controlled trial without randomization)	200 medical students	BJG-EIQ	EI-based intervention reduced academic stress and improved coping skills in medical students.
Rezaei et al., 2023 (35)	USA	Develop an elective for first-year medical students incorporating visual arts instruction and reflective practice to enhance empathy.	Quasi-experimental (controlled trial without randomization)	128 medical students	IRI	Perspective-taking improved significantly, highlighting the role of bias awareness in empathy development.
Kagawa et al., 2023 (43)	Japan	Evaluate how patient storytelling enhances undergraduate medical students’ empathy in Japan over 6 months.	Quasi-experimental (controlled trial without randomization)	159 medical students	JSE	Patient storytelling improved empathy scores immediately and sustained effects for 6 months.
Yuen et al., 2023 (48)	Hong Kong	Assess the impact of a Serious Illness Communication Skills Training (SI-CST) intervention on medical students’ attitudes regarding clinical empathy.	Quasi-experimental (controlled trial without randomization)	185 medical students	JSE	SI-CST enhanced self-efficacy in empathic communication among final-year medical students.
Tariq et al., 2023 (61)	Pakistan	Determine the change in empathy levels of medical students during their professional years and internship.	Quasi-experimental (controlled trial without randomization)	433 medical students	JSPE	A patient-centered module helped sustain empathy scores in third-year medical students.
Lisevick et al.,2023 (59)	USA	Describe a novel medical student leadership development curriculum incorporating core elements of lifestyle medicine.	Quasi-experimental (controlled trial without randomization)	19 medical students	5-Point Likert Scale	Leadership curriculum showed promising results in developing meaningful interpersonal and professional skills.
Pearlman Shapiro et al., 2023 (72)	USA	Investigate preclinical medical students’ experiences volunteering as abortion doulas and its impact on their empathy.	Descriptive, Qualitative	22 medical students		Students’ first exposure to abortion procedures shaped their perceptions of patient care.
Ward et al., 2023 (51)	UK	Introduce a pilot teaching session to prepare second-year medical students for handling difficult conversations.	Quasi-experimental (controlled trial without randomization)	300 medical students		A pilot teaching session improved students’ understanding of managing emotionally challenging consultations.
Leijenaar et al., 2023 (47)	Netherlands	Evaluate a mandatory narrative medicine lesson in a large sample of medical students.	Descriptive, Qualitative	345 total students (203 included in final analysis)		Narrative medicine validated its impact on empathy in medical students in a larger study population.
Erlich et al., 2023 (37)	USA	Assess the impact of medical students completing their own advance directives (ADs) on empathy levels and attitudes toward end-of-life care.	Quasi-experimental (controlled trial without randomization)	548 medical students		Completing ADs deepened reflections on end-of-life care, enhancing clinical empathy and communication.
Donisi et al., 2022 (73)	Italy	Describe the Emoty-Com training and its impact on medical students’ attitudes toward doctors’ emotions.	Observational analytic, Cross sectional (analytic)	264 medical students	EQI	Emoty-Com training improved students’ competence in handling emotional and communication-related worries.
Bętkowska-Korpała et al., 2022 (66)	Poland	Analyze personality characteristics of empathy profiles among medical students, considering personality traits.	Observational analytic, Cross sectional (analytic)	153 medical students	Empathetic Sensitiveness Scale	Medical students clustered into three empathy-based personality groups using the Big Five Model.
Potts et al., 2022 (67)	Multiple	Evaluate the effectiveness of the READ program in improving medical students’ knowledge, attitudes, and skills.	Observational analytic, Cohort	653 medical students	JSE-S	Anti-stigma training improved communication skills and empathy, highlighting intergroup anxiety reduction.
Ardenghi et al., 2022 (74)	Italy	Explore the association of emotional intelligence (EI) and attachment security (AS) with empathy dimensions in medical students.	Observational analytic, Cross sectional (analytic)	253 medical students	IRI	EI mediated the link between AS and empathy, supporting EI training for medical students.
Krishna Bahadur et al., 2022 (75)	Nepal	Evaluate the empathy levels of first-year medical students before and after undertaking a Medical Humanities Module.	Observational analytic, Cross sectional (analytic)	62 medical students	JSE-S	A Medical Humanities module positively impacted student empathy and should be included in curricula.
Lam et al., 2022 (76)	USA	Assess the impact of life story interviews with community-based volunteers on medical students’ empathy development.	Descriptive, Survey (Cross-sectional)	240 medical students		Life story interviews enhanced clinical empathy, reduced burnout, and humanized patient care.
Nandagopal et al., 2022 (34)	USA	Encourage reflective practice by introducing Balint groups to teach students to analyze patient relationships.	Descriptive, Qualitative	60 medical students		Balint groups provided a safe space for students to reflect on challenging clinical experiences.
Ng et al., 2022 (33)	New Zealand	Explore the emotional experiences of fifth-year medical students participating in Balint groups during clinical rotations.	Descriptive, Qualitative	6 medical students		Balint group discussions helped students process clinical encounters and sustain empathy.
Bukowski et al., 2022 (55)	Ireland	Examine the relationship between self-reported empathy and breaking bad news (BBN) communication skills in medical students.	Observational analytic, Cross sectional (analytic)	100 medical students	JSPE-S and EQI	Hospitalization experiences improved students’ empathy by exposing them to patient perspectives.
Epinat-Duclos et al., 2021 (49)	France	Evaluate the impact of Nonviolent Communication (NVC) training on empathy-related skills in medical students.	Experimental, Randomized (parallel group)	312 medical students (123 intervention group, 189 control group)	JSPE and EQ	NVC training improved subjective empathy but did not affect cognitive/emotional empathy scores.
Praharaj et al., 2021 (68)	India	Develop and evaluate the effectiveness of the Stigma, Empathy, and Attitude (SEA) module for improving knowledge and attitudes toward mental illness.	Quasi-experimental (controlled trial without randomization)	157 medical students at baseline, with 66 completing the intervention	MHKS, MICA, JSE, SDS	Post-intervention, mental health knowledge increased, stigma decreased, but empathy scores showed no change.
Fukuyasu et al., 2021 (60)	Japan	Evaluate the impact of the Humanitude Care Methodology on enhancing and sustaining empathy in medical students.	Observational analytic, Cohort	115 medical students; 79 completed all seven test administrations	JSE	Humanitude training improved non-verbal empathy but required reinforcement for long-term effects.
Przymuszała et al., 2021 (50)	Poland	Assess the impact of a modified Group Objective Structured Clinical Experience (GOSCE) on medical students’ communication self-efficacy.	Quasi-experimental (controlled trial without randomization)	126 medical students (80 females, 46 males) participated	EMPATHY Protocol	GOSCE enhanced communication self-efficacy but reduced motivation among female students.
Van Winkle et al., 2021 (52)	USA	Compare the effects of remote learning in 2020 with in-person learning from 2017 to 2019 in a longitudinal observational study.	Observational analytic, Cohort	61 prospective medical students (41 in Colorado, 20 in Utah)	JSE	Remote learning-maintained empathy gains despite engagement challenges in virtual environments.
Yang et al., 2021 (77)	Taiwan	Investigate how preclinical service-learning (SL) experiences affect the development of empathy in medical students.	Observational analytic, Cohort	70 medical students (51% male, 49% female) participated in surveys	JSPE-S	Service-learning positively influenced compassionate care and perspective-taking in medical students.
Airagnes et al., 2021 (78)	France	Explore the relationship between personality traits and cognitive empathy in medical students.	Observational analytic, Cohort	311 medical students	JSE-S	Cognitive empathy improved in medical students regardless of personality type.
Imperato et al., 2021 (36)	USA	Evaluate the effects of reflection exercises on the empathy and emotional intelligence (EI) of third-year medical students.	Descriptive, Survey (Cross-sectional)	285 medical students	JSE	Reflection Rounds prevented empathy decline seen in clerkship students.
Jacoby et al., 2021 (58)	USA	Assess the impact of a novel curriculum (Scholarly Excellence, Leadership Experiences, Collaborative Training [SELECT]) on medical students.	Observational analytic, Cross sectional (analytic)	115 medical students: 39 Year 1 students, 76 Year 2 students	JSE	A novel US medical curriculum maintained stable empathy scores and reduced depersonalization burnout.
Kikukawa et al., 2021 (63)	Japan	Evaluate the impact of a hospitalization immersion experience on medical students’ understanding of patient perspectives and empathy.	Descriptive, Qualitative	488 medical students enrolled; 462 provided complete survey responses		Guided reflective narratives effectively encouraged empathy and reflective learning.
Savitha et al. 2021 (38)	India	Investigate the role of guided reflective narratives on early clinical exposure in first-year medical students.	Descriptive, Qualitative	147 medical students		Empathy gains declined over time, highlighting the need for continued exposure to maintain benefits.
Cecchetti et al., 2021 (54)	Ireland	Assess the effectiveness of a disability education module in fostering long-term empathetic attitudes toward patients with disabilities.	Quasi-experimental (controlled trial without randomization)	320 medical students	JSPE	Learning from patients helped students develop emotional insight and perspective-taking skills.
Rieffestahl et al., 2021 (64)	Denmark	Explore what medical students learn from patients with chronic conditions in communication skills training.	Descriptive, Qualitative	32 medical students		Medical school shaped students’ empathy, with some expressing emotional detachment over time.
Laughey et al., 2021 (39)	UK	Understand the emotional and reflective journey of medical students regarding their relationship with empathy.	Descriptive, Qualitative	20 medical students		Cadaveric dissection was crucial for cognitive and emotional learning, unlike digital anatomy models.
Abrams et al., 2021 (65)	USA	Identify themes from medical students’ reflections on their dissection experience to guide curriculum improvements.	Descriptive, Survey (Cross-sectional)	117 medical students		The Social Emotional Noticing Tool (SENT) improved third-year medical students’ empathetic practice.

#### Quality assessment

The methodological rigor of included studies was assessed using the JBI Critical Appraisal Checklist (23). This validated tool evaluates methodological robustness and minimizes the risk of bias. Authors independently appraised each study based on predefined criteria, including clarity of inclusion criteria, detailed descriptions of study subjects and settings, validity and reliability of exposure measurements, use of standardized condition measurements, identification and mitigation of confounding factors, and appropriateness of statistical analysis. A summary of the quality assessment results is provided in Table 2.

**Table 2:** Quality analysis of the articles using the JBI scale, arranged from the highest to the lowest rating.

Author, year	Q1	Q2	Q3	Q4	Q5	Q6	Q7	Q8	Overall score (/8)
Baseer, 2024 (71)	Y	Y	Y	Y	Y	Y	Y	Y	8
Britz, 2024 (31)	Y	Y	Y	Y	Y	Y	Y	Y	8
Erlich, 2023 (37)	Y	Y	Y	Y	Y	Y	Y	Y	8
Rezaei, 2023 (35)	Y	Y	Y	Y	Y	Y	Y	Y	8
Potts, 2022 (67)	Y	Y	Y	Y	Y	Y	Y	Y	8
Nandagopal, 2022 (34)	Y	Y	Y	Y	Y	Y	Y	Y	8
Epinat-Duclos, 2021 (49)	Y	Y	Y	Y	Y	Y	Y	Y	8
Praharaj, 2021 (68)	Y	Y	Y	Y	Y	Y	Y	Y	8
Yang, 2021 (77)	Y	Y	Y	Y	Y	Y	Y	Y	8
Cecchetti, 2021 (54)	Y	Y	Y	Y	Y	Y	Y	Y	8
Mahmoudi, 2024 (46)	Y	Y	Y	Y	Y	N	Y	Y	7
Arumugam, 2024 (53)	Y	Y	Y	Y	Y	N	Y	Y	7
Rhines, 2024 (57)	Y	Y	Y	Y	Y	Y	N	Y	7
Pearlman, 2023 (72)	Y	Y	Y	Y	Y	N	Y	Y	7
Leijenaar, 2023 (47)	Y	Y	Y	Y	Y	N	Y	Y	7
Bętkowska-Korpała, 2022 (66)	Y	Y	Y	Y	Y	N	Y	Y	7
Ardenghi, 2022 (74)	Y	Y	Y	Y	Y	Y	N	Y	7
Krishna Bahadur, 2022 (75)	Y	Y	Y	Y	Y	N	Y	Y	7
Lam, 2022 (76)	Y	Y	Y	Y	Y	N	Y	Y	7
Van Winkle, 2021 (52)	N	Y	Y	Y	Y	Y	Y	Y	7
Airagnes, 2021 (78)	Y	Y	Y	Y	Y	N	Y	Y	7
Imperato, 2021 (36)	Y	Y	Y	Y	Y	N	Y	Y	7
Kikukawa, 2021 (63)	Y	Y	Y	Y	Y	N	Y	Y	7
Rieffestahl, 2021 (64)	Y	Y	Y	N	Y	Y	Y	Y	7
Kanagasabai, 2024 (42)	Y	Y	Y	Y	Y	N	Y	U	6
Neeley, 2024 (45)	Y	Y	Y	Y	N	N	Y	Y	6
Kadam. 2024 (41)	Y	Y	Y	Y	N	N	Y	Y	6
Khorasani, 2023 (56)	N	Y	Y	Y	Y	N	Y	Y	6
Kagawa, 2023 (43)	Y	Y	Y	Y	N	N	y	Y	6
Yuen, 2023 (48)	Y	Y	Y	Y	N	N	Y	Y	6
Tariq, 2023 (61)	Y	Y	Y	Y	N	N	Y	Y	6
Lisevick, 2023 (59)	N	Y	Y	Y	Y	N	Y	Y	6
Donisi, 2022 (73)	Y	Y	Y	Y	N	N	Y	Y	6
Bukowski, 2022 (55)	Y	Y	Y	Y	Y	N	N	Y	6
Fukuyasu, 2021 (60)	N	Y	Y	Y	Y	N	Y	Y	6
Laughey, 2021 (39)	Y	Y	Y	N	Y	N	Y	Y	6
Abrams, 2021 (65)	Y	Y	Y	Y	Y	N	N	Y	6
Przymuszała, 2021 (50)	N	Y	Y	N	Y	Y	Y	Y	6
Savitha, 2021 (38)	Y	Y	Y	Y	Y	Y	N	N	6
Ng, 2022 (33)	Y	Y	Y	Y	Y	N	N	U	5
Jacoby, 2021 (58)	N	Y	Y	Y	N	N	Y	Y	5
Hashim, 2024 (40)	Y	Y	U	Y	N	N	U	Y	4
Blalock, 2024 (32)	Y	Y	Y	Y	N	N	N	N	4
Ward, 2023 (51)	N	N	Y	Y	N	N	Y	Y	4

#### Justification for narrative synthesis

Although a meta-analysis was initially planned, substantial heterogeneity in outcome measures, study designs, and assessment tools across the included studies precluded the pooling of quantitative data. Given the diversity in methodologies and reporting formats, a statistical meta-analysis was deemed infeasible. Consequently, a narrative synthesis was undertaken, integrating findings through thematic analysis.

#### Thematic analysis approach

This study employed an inductive thematic analysis approach to identify recurring patterns and themes within the extracted data (24). Two reviewers independently analyzed study outcomes, assigning initial codes to descriptive phrases (e.g., “improved empathy scores,” “enhanced self-awareness,” “structured communication training”). The coding process was iterative, with reviewers engaging in regular discussions to refine the coding framework and ensure consistency. Any discrepancies were resolved through consensus.

#### Theme development

Following initial coding, related codes were grouped into broader categories, resulting in the emergence of key themes. The analysis identified the following thematic areas:

- Narrative and Storytelling Interventions
- Reflective Practices and Writing
- Communication Skills Training
- Emotional Intelligence Enhancement
- Experiential Learning and Patient Exposure
- Stress, Burnout, and Coping Interventions
- Assessment Tools and Structural Interventions
- Personalized Interventions and Diversity Considerations

Each theme was critically reviewed and refined until consensus was reached.

#### Theoretical integration

To strengthen the theoretical foundation of this study, the identified themes were mapped onto Daniel Goleman’s model of Emotional Intelligence, which comprises four domains:

- Self-Awareness
- Self-Management
- Social Awareness
- Relationship Management

Reviewers identified the most relevant Goleman domains for each theme based on the reported intervention outcomes. This mapping provided a structured theoretical basis for understanding how various interventions contribute to emotional intelligence development among medical students.

#### Reliability and validation

To enhance the reliability of the thematic analysis, inter-rater reliability was assessed by comparing independently generated codes. The coding and theme development processes were refined iteratively until an acceptable level of agreement was reached. Any remaining discrepancies were resolved through consensus.

#### Presentation of findings

The final themes, along with their corresponding Goleman EI domains and representative studies, were compiled into a comprehensive thematic table. This table (available in Supplementary material) presents each of the 44 studies and illustrates the linkages between intervention types, emergent themes, and theoretical EI domains.

## Results

A total of 44 studies met the inclusion criteria and were analyzed in this systematic review. The included studies varied in terms of study design, intervention type, and outcome measures, reflecting the diverse approaches to EI development in medical education.

### Study selection process

The study selection process is illustrated in Figure 1, following the PRISMA (Preferred Reporting Items for Systematic Reviews and Meta-Analyses) guidelines. Initially, a total of 2,608 records were identified across multiple databases. After removing 206 duplicate records, 2,402 articles proceeded to the screening phase. Based on title and abstract screening, 2,185 articles were excluded for not meeting the inclusion criteria, such as publication before 2021, focus on non-medical student populations, or lack of relevant EI interventions. A total of 217 full-text articles were assessed for eligibility. Of these, 12 articles were inaccessible, leaving 205 studies for full-text review. After applying additional inclusion criteria—specifically, requiring studies to include an EI intervention—the final dataset was refined to 44 articles for qualitative synthesis and thematic analysis.

### Overview of included studies

Table 1 summarizes the included articles, detailing key study characteristics such as author, publication year, intervention type, and primary outcomes. The studies encompassed various EI-focused interventions, ranging from narrative-based approaches to experiential learning and structured communication training.

### Quality assessment

The methodological quality of the included studies was assessed using the JBI Critical Appraisal Checklist. Table 2 presents the results of this quality assessment, highlighting the strengths and limitations of individual studies. The overall quality of the studies varied, with most demonstrating rigorous methodological designs, while some exhibited limitations in blinding procedures and control measures.

### Thematic analysis and mapping to Goleman’s EI model

Eight primary themes were identified from the included studies through an inductive thematic analysis. These themes were then mapped onto Goleman’s Emotional Intelligence domains to establish a theoretical framework for understanding the impact of different interventions. Table 3 displays this thematic mapping, illustrating how specific interventions align with key EI competencies, including self-awareness, self-regulation, social awareness, and relationship management. The most frequently targeted EI domain across studies was empathy, followed by self-awareness and social skills, whereas self-regulation and motivation were less frequently addressed.

**Table 3:** Thematic analysis based on Goleman’s EI domains.

Theme	Linked Goleman EI domain(s)	Description	Representative studies (References)
1. Narrative and Storytelling Interventions	Empathy, Self-Awareness, Social Skills	Interventions using patient stories, narrative medicine, or story reading are designed to enhance empathy by allowing students to see experiences from others’ perspectives while fostering self-reflection and interpersonal understanding.	Mahmoudi et al. (46), Kanagasabai et al. (42), Kagawa et al. (43), Lam et al. (76), Leijenaar et al. (47), and Pearlman Shapiro et al. (72)
2. Reflective Practices and Writing	Self-Awareness, Self-Regulation, Empathy	Reflective writing and guided narrative practices encourage deep self-examination and emotional regulation, which are key for both recognizing and managing one’s own emotions and empathizing with others.	Kadam et al. (41), Rezaei et al. (35), Hashim et al. (40), Imperato and Strano-Paul (36), Nandagopal and Walker (34), Ng et al. (33), and Savitha et al. (38)
3. Communication Skills Training	Effective Communication (Social Skills), Empathy	Structured training modules (such as the EMBRACE module, SI-CST, and GOSCE) focus on improving interpersonal communication and empathetic interaction, directly supporting students’ ability to connect with patients.	Arumugam et al. (65), Yuen et al. (48), Przymuszała et al. (50), and Ward and Howick (51)
4. Emotional Intelligence Enhancement	Self-Regulation, Motivation, Empathy, Self-Awareness, Social Skills	Interventions explicitly targeting EI (e.g., cognitive fitness or Humanitude training) aim to improve overall emotional regulation and drive, thereby enhancing a range of EI components.	Rhines et al. (57), Khorasani et al. (56), Fukuyasu et al. (60), Jacoby et al. (58), and Ardenghi et al. (74)
5. Experiential Learning and Patient Exposure	Empathy, Social Skills, Self-Awareness	Hands-on clinical experiences, including patient exposure and practical exercises like cadaveric dissection or hospitalizations, deepen understanding of patient perspectives and enhance interpersonal skills.	Kikukawa et al. (63), Abrams et al. (65), Pearlman Shapiro et al. (72), and Rieffestahl et al. (64)
6. Stress, Burnout, and Coping Interventions	Self-Regulation, Motivation	Programs that address stress, burnout, and coping strategies work to enhance self-regulatory skills and sustain motivation, ultimately supporting better emotional management in challenging clinical environments.	Khorasani et al. (56), Lam et al. (76), Jacoby et al. (58), and Ardenghi et al. (74)
7. Assessment Tools and Structural Interventions	Effective Communication, Social Skills, Empathy (through feedback mechanisms)	The use of tools (like the Social Emotional Noticing Tool) and systemic approaches to measuring and reinforcing EI reflect an integrated framework that supports all EI domains by providing structured feedback and evaluation.	Blalock et al. (32) and Britz et al. (31)
8. Personalized Interventions and Diversity Considerations	Self-Awareness, Empathy, Motivation	Tailored interventions that consider individual differences (e.g., gender, personality profiles, or intergroup dynamics) help optimize the development of empathy and related EI aspects by addressing unique learner needs.	Praharaj et al. (68), Bętkowska-Korpała et al. (66), and Potts et al. (67)

### Quantitative data and supplementary analysis

While the majority of studies focused on qualitative outcomes, several provided quantitative data measuring changes in EI competencies using standardized tools. Supplementary Table 1 outlines the studies that included quantitative assessments, highlighting reported improvements in emotional intelligence scores post-intervention. However, due to significant heterogeneity in outcome measures and intervention types, a meta-analysis could not be conducted. Instead, a narrative synthesis was employed to integrate findings.

### Comprehensive thematic table

To further consolidate the findings, a detailed thematic table was created, linking specific study interventions with Goleman’s EI domains. Supplementary Table 2 provides this comprehensive thematic analysis, offering insights into the distribution of interventions across different emotional intelligence competencies. This table serves as a valuable resource for identifying patterns in EI training and intervention efficacy across medical education curricula.

### Summary of key findings

The analysis revealed that storytelling and narrative-based interventions were particularly effective in fostering empathy, while communication skills training and experiential learning contributed significantly to improved social awareness and interpersonal communication. Reflective practices and structured emotional intelligence enhancement programs demonstrated positive effects on self-awareness and emotional regulation. The integration of multi-component approaches—combining direct patient interactions, structured training modules, and guided reflections—yielded the most promising outcomes in EI development.

## Discussion

Recent research in educational psychology has provided substantial evidence supporting the pivotal role of EI in achieving success in higher education. Beyond cognitive intelligence, often measured by intelligence quotient (IQ), EI enables individuals to recognize, understand, and regulate emotions—both their own and those of others. Studies indicate that higher levels of EI are positively correlated with improved academic performance, greater student engagement, and enhanced overall well-being. Consequently, integrating strategies to cultivate key EI competencies, such as empathy, self-awareness, and relationship management, has become essential for modern educators and learners striving to achieve both academic and personal growth.

Researchers highlight EI as a key academic competency, emphasizing its role in resilience, commitment, and success, with evidence showing that students with higher EI levels are more engaged in class attendance, studying, and faculty interactions (25). Similarly, research has demonstrated a strong correlation between higher EI, stronger social relationships, and greater well-being (26). Furthermore, research has shown that students with higher EI tend to achieve better GPAs and higher graduation rates compared to those with lower EI. Additionally, researchers suggest that EI is a strong predictor of student engagement, which contributes to improved academic performance, personal development, and overall university satisfaction (27).

Expanding on this perspective, researchers argue that students with stronger EI possess better resources to navigate academic challenges successfully (28). Additionally, their research suggests that personality traits, particularly extroversion, may also contribute to academic achievement. However, not all studies align with these conclusions. García et al. (29) report no significant relationship between EI and academic performance, which challenges the prevailing assumption of a direct correlation. While this finding may seem contradictory, it is essential to differentiate between academic performance and academic achievement. The former primarily focuses on final outcomes, whereas the latter considers a student’s progress throughout the learning process. Rodríguez et al. (30) also question whether EI directly influences academic performance. Their study, conducted among universities in Spain, found that high levels of substance use among participants might have obscured the potential benefits of EI in academic success.

Recognizing the importance of EI in higher education, the current study advocates for integrating EI development into academic curricula. This study supports that higher EI levels contribute not only to academic success but also to enhanced leadership skills and overall well-being. Furthermore, educators play a pivotal role in fostering students’ social, emotional, and intellectual growth. Given their influence on student success, evaluating the EI competencies of educators is of paramount importance.

In addition to its relevance in traditional academic settings, recent studies have explored the ethical implications of EI in the context of Artificial Intelligence (AI) and e-learning. By examining the potential risks and benefits of integrating AI-driven educational tools, researchers aim to guide policymakers, educators, and AI developers toward responsible implementation. The objective is to ensure that technological advancements in education prioritize both efficiency and human well-being. This innovative approach seeks to shape future research endeavors, ultimately enriching the educational landscape through a balanced integration of EI and technology.

Researchers have sought to expand the existing body of EI models by evaluating the validity of novel assessment algorithms. However, despite the establishment of robust EI measurement tools, the development of empirically validated interventions remains an area requiring further research. This article analyzed 44 studies, using Daniel Goleman’s EI model as the theoretical underpinning for the thematic analysis.

### Assessment tools and structural interventions

A 2024 German study by Britz et al. (31) examined the comparative effectiveness of simulated patient-based communication training versus real patient-based training for medical students. The study utilized self-reported measures such as the Jefferson Scale of Physician Empathy (JSPE) and the Interpersonal Reactivity Index (IRI), alongside the Consultation and Relational Empathy (CARE) scale, which was administered by simulated patients. While self-reported empathy scores (JSPE, IRI) showed no significant differences between the two groups, students who interacted with simulated patients received higher patient-reported empathy scores on the CARE scale than those who engaged with real patients. One possible explanation for this discrepancy is the heightened anxiety experienced by students when interacting with real patients, underscoring the necessity of a progressive training model that gradually transitions students from simulated to real-patient encounters following adequate preparatory training (31).

Similarly, a study conducted at Michigan State University explored the role of observational skills in fostering empathy (32). The researchers introduced the Social Emotional Noticing Tool (SENT) to third-year medical students during their family medicine rotations, encouraging them to actively recognize and reflect on the presence—or absence—of empathy in clinical interactions. Their findings indicated that students who were exposed to positive role models demonstrated greater engagement in empathetic interactions, whereas certain clinical environments negatively influenced students’ ability to sustain empathy. These results highlight the critical role of faculty and mentors in shaping empathetic behavior during clinical training.

The barriers noted by Blalock et al. align with the findings of Britz et al., particularly regarding the challenge of balancing observation with real-time engagement. The studies suggest that students may struggle with real-patient encounters due to a lack of prior experiential training (31, 32). Additionally, while self-reported empathy assessments provide insights into students’ perceived strengths and weaknesses, they may also lead to an overestimation of perceived empathy gains post-intervention. Therefore, incorporating objective empathy feedback, as was done in Britz et al. (31), may yield a more accurate assessment of students’ abilities to meaningfully engage with patients. Furthermore, long-term follow-up assessments may be necessary to determine the extent to which these interventions contribute to sustained behavioral changes in clinical practice.

### Reflective practices and writing

Reflection and empathy are inherently connected, making reflective practices an essential tool for fostering empathetic engagement in medical education. One well-documented intervention is the use of Balint groups, which have been extensively studied for their role in enhancing empathy among medical students. Traditionally utilized in family medicine, Balint groups offer a structured, small-group setting where participants discuss clinical cases and explore their emotional responses to patient interactions (33). For preclinical students, these sessions provide an opportunity for self-reflection, enabling them to identify potential biases, broaden their perspectives, and develop a more nuanced understanding of patient care (34).

A study explored the impact of visual arts education as an elective for medical students, demonstrating improvements in observational and communication skills. Students reported that reflective practices facilitated greater empathy in patient interactions (35). Furthermore, research suggests that consistently employing these sessions throughout clinical training may increase empathy levels and prevent the decline of empathy that is often observed as students progress through their training (36).

A particularly relevant application of reflective practice is in end-of-life (EOL) care. Erlich et al. (37) investigated an intervention in which medical students completed their own Advanced Directive, accompanied by a written reflection on the anticipated effects of this experience on their future clinical practice. This intervention was found to significantly enhance students’ understanding of EOL care, emphasizing the value of experiential learning in emotionally complex areas of medicine.

Reflective practices also extend beyond patient interactions to encompass relationships among medical professionals. A study by Savitha et al. (38) highlighted the importance of reflective writing during early clinical education (ECE) sessions. Through structured reflections, students demonstrated an improved ability to empathize with patients and caregivers and fellow clinicians and healthcare staff, reinforcing the significance of professionalism and teamwork in medical practice.

An innovative approach to reflective writing was introduced by Laughey et al. (39), who prompted students to compose “Love and Breakup Letters” following encounters with standardized patients (SPs). These letters examined three key themes: empathy as art and artifice, empathy as a burden, and empathy as a virtue. Notably, the study identified frequent references to “fake empathy,” particularly in the context of OSCEs, where students may feel compelled to demonstrate empathy artificially. This suggests that reflective practices may be most effective when applied during clinical years, where students engage with real patients in authentic medical settings.

Although empathy scores did not consistently show statistically significant improvements post-intervention, reflective practices have been shown to elicit strong emotional responses, particularly in cases where patients received inadequate care. Additionally, reflective writing facilitated empathy toward patients, colleagues, and the broader medical community (40). Kadam et al. (41) argue that the benefits of reflective writing may take time to materialize, requiring longitudinal evaluation to assess sustained impacts. Moreover, they emphasize the importance of providing students with structured feedback on their reflective writing to maximize its benefits. Given these findings, integrating reflective practices into medical education, particularly over an extended period, warrants further consideration to reinforce the emotional and ethical dimensions of clinical practice.

### Narrative and storytelling interventions

Narrative and storytelling interventions have been proven effective tools for enhancing EI and empathy among medical students. These methods utilize personal experiences, reflective writing, and patient narratives to deepen understanding of patient experiences and emotional states. Kanagasabai et al. (42) used storytelling from women with abnormal uterine bleeding to emphasize the importance of empathetic communication in gynecological consultations, which requires a sensitive and compassionate approach. This resulted in improved empathy and non-judgmental communication among students. Similarly, Kagawa et al. (43) demonstrated that patient storytelling regarding chronic illness significantly impacted medical students’ empathy levels. The intervention of storytelling demonstrates the power of medical professionals’ words on patient well-being and experience. These interventions provide students with a more profound understanding of patient perspectives, enabling them to recognize their own emotions and respond more compassionately in clinical interactions.

Historically, fictional and non-fictional storytelling have been used to elicit specific emotions and reinforce universal lessons meant to be learned from narrations. This has led to other artistic modalities being considered for their ability to evoke emotions and self-reflection, such as paintings and movies. These can also be generalized to medical practice and education, with research suggesting that narrative medicine interventions using books and films encouraged reflection on patient-centered themes, thereby fostering authentic patient engagement (44). Studies have shown that storytelling interventions, including narrative reading, video simulations, and art-based observation techniques, significantly increase empathy scores among medical students. Neeley et al. (45) implemented an empathy and visual arts curriculum during a pediatrics clerkship which successfully fostered empathic behaviors by teaching observational skills to identify patient emotions. Other authors have also concluded that story reading improved general empathy in medical students, including cognitive and emotional empathy (46). Consistent improvement in empathy scores across several studies utilizing narration and storytelling highlights the importance of incorporating these methodologies into medical curricula to enhance emotional intelligence and patient care (44–47).

### Communication skills training

Medical schools increasingly recognize the importance of equipping students with practical communication skills, as these competencies directly enhance empathy and contribute to higher emotional intelligence (48). Empathic communication is considered a skill that can be developed through structured training, enabling future physicians to, understand patients’ psychological states better and make informed clinical decisions. However, methods for teaching these skills vary across institutions, making it essential to establish evidence-based approaches to integrating communication training into the medical curriculum.

Several studies have explored different approaches to communication skills training. Research has demonstrated that nonviolent communication training significantly enhances self-perceived empathy, supporting its potential as a valuable addition to medical education (49). Another widely studied method is the Group Objective Structured Clinical Experience (GOSCE), which allows students to develop communication skills by navigating complex patient interactions in a structured setting (50). This hands-on approach may be particularly beneficial for students who learn best through direct engagement. Similarly, other medical schools have expanded interactive training methods, incorporating pre-reading materials on physician-patient conflicts, video analyses of real consultations, and role-playing exercises with standardized patients (51). Such interactive models have been shown to improve students’ ability to handle difficult conversations, reinforcing the value of experiential learning. Despite logistical challenges, remote learning strategies implemented during the COVID-19 pandemic demonstrated that communication skills training can still effectively enhance teamwork, empathy, and bias mitigation among medical students (52).

In addition to general communication training, specialized programs have been developed to prepare students for emotionally challenging conversations. Two widely recognized models are SPIKES and EMBRACE (53). The SPIKES model is a structured framework guiding physicians through difficult conversations by incorporating setting, perception, invitation, knowledge, and empathy, ensuring that distressing information is sensibly conveyed. The EMBRACE model builds on this approach by providing structured training in breaking bad news with compassion, filling a gap in traditional medical education. Both models highlight the need for structured, practical training in emotionally challenging communication to improve doctor-patient relationships and healthcare outcomes.

While these interventions show promise, there are notable challenges in ensuring that students retain and apply these skills throughout their careers. Although training programs can enhance empathy and communication in the short term, sustaining these improvements over the long term remains difficult. Structural barriers within medical education—such as emotional burnout, a culture emphasizing efficiency over patient connection, and the need for emotional self-protection—can erode students’ ability to maintain empathetic interactions (54). To address these challenges, continuous reinforcement of communication skills, along with institutional support for stress management and emotional resilience, is crucial.

Another limitation of traditional communication skills training is the lack of explicit social skills development. Research suggests that social competencies, as measured by emotional intelligence scales, are strongly linked to physicians’ ability to effectively deliver difficult news (55). However, social skills are inherently complex and difficult to teach in a standardized manner. Tailored, student-specific training that accounts for individual learning styles and skill levels may offer a potential solution, ensuring that communication training is more adaptable and effective in fostering long-term empathic engagement.

### Emotional intelligence enhancement

This review indicates that EI is essential for academic success and the development of critical competencies such as communication, empathy, stress management, and patient care. A study by investigating the impact of an EI-based training program, combined with consulting support, demonstrated that the intervention significantly enhanced EI components while reducing academic stress and students’ responses to stressors (56).

Our review suggests that EI can be cultivated through structured interventions, including EI training programs, reflective practices, and mindfulness exercises. Several studies reinforce the importance of incorporating these strategies into medical education. For example, Rhines et al. (57) demonstrated the feasibility of embedding a cognitive fitness program into existing curricula without negatively affecting academic performance. By utilizing a structured Cognitive Fitness Program, their study observed significant improvements in medical students’ focus, gratitude, and resilience.

The literature further supports the notion that EI can be improved through targeted training programs. Jacoby et al. (58) developed a novel EI-based curriculum for medical students, reporting a significant reduction in empathy decline following the intervention. Additionally, Lisevick et al. (59) focused on leadership development within medical education, successfully integrating EI components into existing programs. Their findings indicated increased confidence across a range of professional competencies, as measured through a Likert scale assessment.

Further evidence highlights the effectiveness of workshops and lectures in fostering EI development among medical students. Fukuyasu et al. (60) found that participation in the Humanitude Care Methodology program led to significant improvements in students’ Jefferson Scale of Empathy (JSE) scores. Collectively, these findings underscore the value of integrating EI-focused interventions into medical training, reinforcing the need to develop emotional intelligence as an essential skill for medical students to improve both their professional effectiveness and well-being.

### Experiential learning and patient exposure/stress, burnout, and coping interventions

EI is increasingly recognized as a crucial component of medical education, leading to the implementation of various interventions aimed at enhancing EI-related competencies among medical students. Several studies have explored different experiential approaches to improving EI, including patient-centered learning, immersive simulations, and structured reflective practices.

One effective intervention involves patient-centered modules and workshops. Tariq et al. (61) implemented a patient-centered module at the start of clinical exposure in the third year of medical school, incorporating reflective writing, role play, humanities, and communication skills. This intervention significantly improved empathy scores among students. Additionally, the study incorporated a stress management workshop in the final year to help students identify stressors and practice coping strategies, which contributed to improved empathy scores during their internship. Similarly, Schweller et al. (62) found that reflective activities helped enhance short-term empathy in medical students, emphasizing the importance of self-reflection in EI development.

Experiential learning through simulated patient encounters has also been shown to enhance EI. Kikukawa et al. (63) conducted a study where fifth-year medical students participated in a two-day, one-night hospitalization experience course. This intervention provided students with firsthand insights into patients’ perspectives, distress, and anxiety—not only from their own simulated experiences but also through observing and communicating with inpatients. The authors concluded that this approach was effective in strengthening empathy by improving students’ understanding of patients’ emotional and psychological states.

Training in communication skills with real patients, particularly those managing chronic illnesses, has demonstrated significant benefits in developing EI. Rieffestahl et al. (64) examined a mandatory communication course for fourth-year medical students, where students interacted with patients diagnosed with conditions such as COPD, heart disease, and diabetes. These encounters enabled students to gain a deeper understanding of illness from the patient’s perspective, recognize the diverse needs of patients, and appreciate the importance of aligning doctors’ and patients’ expectations. These authentic patient interactions provided students with valuable opportunities to engage emotionally, reinforcing the role of EI in effective clinical communication.

Another innovative approach to fostering EI involves reflective writing exercises centered around cadaveric dissection experiences. Abrams et al. (65) introduced such an intervention, which promoted both personal and professional development while fostering resilience and a sense of connection among students. The reflective writing component captured themes related to gratitude, humanism, compassion, empathy, and mortality—key elements essential for developing EI in clinical practice.

Overall, these experiential learning interventions have yielded positive outcomes, with multiple studies reporting improvements in students’ empathy, self-awareness, and communication skills. Additionally, they have contributed to the development of professional identity and values essential to medical practice. However, some studies highlight challenges in sustaining long-term empathy gains without continued reinforcement. Students sometimes struggled to balance the emotional demands of patient interactions with professional detachment, underscoring the need for ongoing support and structured guidance throughout medical education.

### Personalized interventions and diversity considerations

Similar to Blalock et al., who examined how the skill of noticing supports empathy in medical students, Bętkowska-Korpała et al., explored how awareness of students’ innate empathy capacity, shaped by personality traits, can inform the design of more effective training interventions (32, 66). The intervention required students to analyze their psychological functioning styles by assessing their personality and empathy traits using the Personality Inventory (NEO-PI-R) and the Empathic Sensitiveness Scale (ESS). The findings showed that some students naturally exhibited strong empathetic engagement, while others relied on a more cognitive, analytical approach, and some experienced high emotional distress when exposed to patient suffering. These differences highlight the necessity of tailoring empathy training based on students’ psychological traits, ensuring that interventions support emotional resilience.

The “Responding to Experienced and Anticipated Discrimination (READ)” program used in a study by Potts et al. (67), developed and implemented across 10 countries, builds on this notion for personalized empathy training. Participants were evaluated using various assessment tools, including the Mental Health Knowledge Schedule (MAKS) to assess mental health knowledge, the Mental Illness: Clinician’s Attitudes (MICA) scale to measure attitudes toward mental illness, the Social Distance Scale (SDS) to evaluate stigma and willingness to interact with individuals with mental illness, and the Jefferson Scale for Empathy (JSE) to assess self-reported empathy. The program also integrated simulated patient assessments, using the Jefferson Scale of Patient Perception of Physician Empathy (PPE) to provide post-intervention evaluations. The study found that the intergroup interactions significantly improved students’ knowledge, attitudes, and clinical communication skills when addressing mental illness stigma while reducing prejudice and intergroup anxiety, allowing students to engage more empathetically with patients with mental illness (67).

A similar intervention, the Stigma, Empathy, and Attitude (SEA) educational module, was implemented in a study from Praharaj et al. (68) to improve medical students’ knowledge and attitudes toward individuals with mental illness. While the SEA module, like the READ program, successfully enhanced mental health knowledge and reduced negative attitudes, it did not lead to measurable improvements in empathy scores. A key distinction is that the READ program incorporated objective assessments from simulated patients, whereas the SEA module relied exclusively on self-reported empathy scores. This suggests that while self-reported empathy assessments can be useful, students may overstate their perceived empathy gains following an intervention. Thus, incorporating objective empathy feedback, as seen in the READ program, may offer a more accurate evaluation of medical students’ ability to engage meaningfully with patients.

These findings highlight the need for a combination of structured and personalized empathy training. Personality-based interventions, support students in understanding their emotional tendencies, while interactive programs like READ underscore the importance of engaging with real or simulated patients in developing practical empathetic skills. The lack of empathy improvement in the SEA module underscores the limitations of passive learning, reinforcing the importance of experiential training in medical education.

### Barriers and contextual considerations

While numerous interventions reviewed demonstrated promising outcomes, it is crucial to consider that emotional intelligence development does not occur in a vacuum. Individual and contextual factors can act as barriers to the efficacy of even the most well-designed programs. Students’ prior educational experiences, early emotional socialization, and cultural background can significantly influence their engagement with and response to EI interventions. For instance, students raised in emotionally restrained environments or cultures that discourage open expression may find reflective practices or narrative exercises less accessible. Additionally, educational systems that prioritize academic performance over socio-emotional learning may inadequately prepare students for EI-based activities (69). These nuances must be considered when designing and implementing EI curricula to ensure equity, cultural responsiveness, and effectiveness across diverse student populations.

Despite increasing recognition of the importance EI in enhancing clinical competence, communication, and resilience, several intrinsic and extrinsic barriers hinder its development in medical learners. Two underexplored dimensions, including neurodiversity and aging-related cognitive changes, present unique challenges that warrant greater integration into medical education frameworks. Neurodiverse individuals, including those with autism spectrum disorder, Attention-Deficit/Hyperactivity Disorder or other cognitive variations, may face inherent difficulties in interpreting social cues, managing interpersonal affect, or engaging in reflective emotional processing. Traditional EI training often assumes a neurotypical baseline, overlooking alternative emotional processing styles and communicative preferences. For example, learners with ASD may struggle with tasks involving emotional inference or empathy modeling, not due to a lack of compassion, but because of different neurocognitive pathways in social cognition (70). Consequently, standardized EI curricula risk marginalizing neurodiverse learners by failing to provide inclusive scaffolding or multimodal approaches that accommodate varied emotional landscapes.

Simultaneously, aging medical students or mid-career professionals returning for advanced training may also experience EI-related barriers linked to cognitive aging. Age-related changes in prefrontal cortical regulation can affect emotional regulation, working memory, and cognitive flexibility which are functions that underpin adaptive emotional responses in complex clinical environments. Moreover, older learners may have entrenched coping mechanisms or prior cultural conditioning that conflict with contemporary approaches to emotional self-awareness or vulnerability in training environments.

An important contextual factor that may influence the effectiveness of EI interventions is the underlying curricular design specifically is whether the program follows a student-centered, problem-based approach or a more traditional, lecture-based structure. Student-centered curricula often place greater emphasis on self-directed learning, peer collaboration, reflective practice, and early clinical exposure all of which align closely with EI competencies such as self-awareness, social awareness, and relationship management. In contrast, conventional curricula may not prioritize these affective domains to the same extent, potentially limiting opportunities for sustained EI development. Therefore, curriculum type may act as a confounding variable in interpreting intervention outcomes and should be accounted for when designing and evaluating EI programs in medical schools.

Together, these dimensions underscore that EI is not uniformly accessible or trainable across all learner profiles. Recognizing the impact of neurodiversity and aging on EI development invites the need for differentiated instructional strategies, inclusive curriculum design, and greater pedagogical flexibility. By doing so, medical education can foster not only technical competence but also a more equitable and emotionally resilient clinical workforce.

### Limitations of the study

This study is subject to several limitations. Inclusion of studies published exclusively in English may have resulted in the exclusion of relevant findings from non-English literature, potentially limiting the generalizability of the results. The heterogeneity in study designs, intervention types, and outcome measures across the included studies presents a challenge in drawing direct comparisons and aggregating quantitative data, necessitating a narrative synthesis rather than a meta-analysis. While Goleman’s model was selected for its practical utility and alignment with behavioral competencies relevant to medical training, it is important to acknowledge the limitations associated with this framework. Scholars have raised concerns regarding its overlap with personality traits, particularly dimensions of extraversion and conscientiousness (8). Additionally, the model has faced critiques for its conceptual ambiguity and inconsistent operationalization across studies. Unlike the ability-based EI model developed by Mayer and Salovey which emphasizes EI as a distinct cognitive capacity, Goleman’s mixed model incorporates a broader range of socio-emotional traits, which may dilute predictive power in some educational environments. Future systematic reviews may benefit from a comparative framework that integrates multiple EI models to yield more nuanced conclusions and support targeted curriculum development.

Additionally, variations in the measurement tools used to assess EI and empathy may introduce inconsistencies in reported outcomes. Another key limitation is the reliance on self-reported measures in many of the included studies, which may be subject to social desirability bias, potentially inflating the effectiveness of interventions. Furthermore, while many studies demonstrated short-term improvements in EI-related competencies, long-term follow-up data were often lacking, making it difficult to assess the sustainability of intervention effects. While numerous studies have demonstrated short-term improvements in EI-related competencies following targeted interventions, a critical gap remains in assessing their long-term impact. Without comprehensive follow-up data, it is challenging to determine whether these gains are sustained throughout medical training and into clinical practice. The transient nature of these improvements raises concerns about the durability of EI-focused educational strategies and their ability to effect lasting behavioral change.

Future research should focus on developing standardized, empirically validated interventions that incorporate long-term follow-ups to assess the sustainability of EI improvements. Additionally, more rigorous experimental designs, such as randomized controlled trials, are needed to strengthen causal inferences regarding the effectiveness of EI interventions. The integration of multimodal assessment approaches, combining self-reported measures with objective assessments and observational data, could further enhance the reliability of findings. Lastly, given the increasing role of digital learning in medical education, future studies should explore how technology-based EI interventions, including virtual reality simulations and AI-driven emotional intelligence training, can complement traditional teaching methods to foster long-term improvements in EI competencies among medical students.

Future research should also examine the role of curriculum design in shaping the success of EI interventions. Comparative studies evaluating EI development within student-centered versus traditional curricula may yield important insights into which educational models best support emotional competence. This line of inquiry could help tailor EI strategies to specific curricular frameworks and ensure that training is optimally aligned with institutional learning cultures and pedagogical philosophies.

## Conclusion

In conclusion, various interventions have demonstrated significant potential in enhancing emotional intelligence among medical students, yielding positive outcomes in personal well-being, interpersonal communication, and professional development. The most effective approach involves a multifaceted, longitudinal integration of EI-focused strategies throughout the medical curriculum, beginning early and reinforced consistently across clinical years. Combining empathy-focused activities, real patient interactions, standardized patient encounters, mindfulness-based stress reduction, and guided peer discussions fosters self-awareness, emotional regulation, and stress management. Repeated practice and reflection, coupled with faculty mentorship and debriefing sessions, further solidify these skills. The broader implications of cultivating EI in medical education extend to improved doctor-patient relationships, enhanced teamwork, reduced burnout, and better clinical decision-making—factors that ultimately contribute to higher patient satisfaction and more compassionate healthcare delivery. Future research should explore the most effective combinations of interventions and their long-term impact across various stages of medical training and professional practice.

## Data Availability

The original contributions presented in the study are included in the article/Supplementary material, further inquiries can be directed to the corresponding author.
