# Supplementary material for: Enhancing emotional intelligence in medical education: a systematic review of interventions
Source: Front Med (Lausanne). 2025 Jul 28;12:1587090. doi: 10.3389/fmed.2025.1587090 (PMC12336118; doi:10.3389/fmed.2025.1587090)
Supplement: Supplementary file 1 [file Data_Sheet_1.docx]

**Supplementary Table 1** Represents the studies that contained qualitative data.

| **Author, Year** | **EI Scale used** | **Mean (before intervention)** | **SD or SE (before intervention)** | **Minimum reported score (before intervention)** | **Maximum reported score (before intervention)** | **Mean (after intervention)** | **SD or SE (after intervention)** | **Minimum reported score (after intervention)** | **Maximum reported score (after intervention)** | **Population (n)** | **P-value (or anything that tells us about significance)** | **Control groups? (if so, what were the values compared to control)** |
| --- | --- | --- | --- | --- | --- | --- | --- | --- | --- | --- | --- | --- |
| Baseer, 2024 | JSPE |  |  |  |  | 0.75 | 17.6 |  |  | 120 |  |  |
| Kanagasabai, 2024 | JSPE | 0.143 0.188 |  |  |  |  |  |  |  |  |  |  |
| Rhines, 2024 | CF | 126.4 | 24.1 |  |  | 146.5 | 25.6 |  |  | 80 | <0.0001 |  |
| Kadam, 2024 | Bar-on Emotional Quotient Inventory | 130.56 | 16.45 |  |  | 140.78 | 15.67 |  |  | 120 |  |  |
| Khorasani, 2023 | BJG-EIQ | 21.29 26.76 | 6.39 7.93 |  |  | 24.04 29.95  24.30 29.40 | 6.33 7.60  6.25 7.76 |  |  | 65 | 0.01 0.02 | 0.95 0.81  0.041 0.035  0.042 0.044 |
| Rezaei, 2023 | IRI | 19 | 4.3 |  |  | 20.2 | 4.5 |  |  | 128 | <0.0125 |  |
| Kagawa, 2023 | JSE-S | 107.9 109.3 106.9 | 11.7 10.8 12.22 |  |  | 111.2 112.7 110.2 | 11.3 10.3 11.9 |  |  | 159 | 0.4 0.5 0.4 |  |
| Yuen, 2023 | JSE | 111.38 | 9.06 |  |  | 113.87 113.92 | 0.34 11.17 |  |  | 185 | 0.01 |  |
| Tariq, 2023 | Jefferson Scale of Physician Empathy | 4.24 4.54 4.21 4.22 4.39 | 0.35 0.75 0.65 0.42 0.44 |  |  |  |  |  |  | 70 | 0.04 0.11 >0.99 0.27 4.39 |  |
| Lisevick, 2023 | 5 point Likert Scale | 3.12 3.06 2.55 |  |  |  | 4.2 4.00 3.55 |  |  |  | 19 | <0.001 <0.001 <0.001 |  |
| Donisi, 2022 | NA | 4.0 3.3 5.6  5.4 4.5 6.0 | 2.2 2.4 2.4 2.2 2.6 2.3 | 3.3 | 6 | 3.7 3.4 5.1 5.4 4.0 5.2 | 2.0 2.0 2.0 2.1 2.2 2.2 | 3.4 | 5.4 | 106 140 107 140 107 140 | 0.9 0.2 0.8 0.03 0.0 0.03 |  |
| Bętkowska-Korpała, 2022 |  |  |  |  |  |  |  |  |  |  |  |  |
| Ardenghi, 2022 | IRI |  |  |  |  | 2.67  2.82 2.47 1.45 1.52 1.36 2.71 2.74 2.66 91.32 91.87 90.66 29.32 28.76 29.84 53.79 53.37 54.34 52.61 54.09 50.73 0 -0.10 0.13 | 0.62 0.57 0.63 0.67 0.65 0.69 0.71 0.73 0.68 3.72 13.00 14.63 6.15 6.06 6.23 11.72 11.72 11.75 12.03 12.15 11.65 1.52 1.50 1.54 | 0.86 0 0.71 46 11  28  15  -4.79 | 4 3.29 4 124 45.71 85 81 3.80 | 253 | 0.17 0.008 0.24 0.001 -0.33 0.033 |  |
| G C Krishna, 2022 | JSE-S | 106.5 | 10.35 | 103.12 103.13 8.74 | 109 108.07  59.81 | 115.58 | 8.8 | 111.54  116.29  8.1 | 117.75 116.44 61.84 | 62 | 0.143 0.100 <0.001 <0.001 <0.001 0.096 <0.001 0.017 0.0013 0.005 0.054 |  |
| Lam, 2022 | CARE | 3.86 | 0.78 | 3.7 | 4 | 4.18 | 0.68 | 4.1 4.1 | 4.3 | 60 | <0.001 <0.001 0.01 0.06 0.02 |  |
| Olsen, 2022 | IRI | 23.5 | 4.27 |  |  | 23.84 | 4.55 |  |  | 8255 | <0.01 | 0.01 0.05 |
| Epinat-Duclos, 2021 | JSPE | 31.14 | 0.33 |  |  | 32.09 | 0.43 |  |  | 122 67 | 0.02 | 30.86 0.33 30.73 0.33 0.7 |
| Praharaj, 2021 | JSE-S | 86.7 85.9 | 6.9 6.6 |  |  | 87.9 89.3 | 7.1 7.7 |  |  | 66 38 | 0.23 |  |
| Fukuyasu, 2021 | JSE | 109.9 | 11.9 |  |  | 112.9 114.7 | 12.3 14.3 |  |  | 115 89 | < 0.01  0.001 |  |
| Przymuszała, 2021 | Self-efficacy scale | 3.79 | 0.87 |  |  | 4.05 | 0.71 |  |  | 126 | 0.043 0.005 <0.001 0.002 |  |
| Van Winkle, 2021 | JSE | 109 |  |  |  | 112 |  |  |  | 60 | 0.003 |  |
| Yang, 2021 | JSPE-S |  | 0.934 |  |  |  | 1.121 |  |  | 70 65 |  |  |
| Airagnes, 2021 | JSE-S | 109.81 | 11 |  |  | 110.22 | 10.19 |  |  | 311 | 0.496 |  |
| Imperato, 2021 | JSE-S | 80.4 | 6.7 |  |  | 82.6 | 8.1 |  |  | 185 120 | 0.012 |  |
| Jacoby, 2021 | JSE | 117.72 | 11.03 | 115.74 | 119.93 | 118.33 115.42 | 14.64 13.32 | 115.42 | 118.33 | 39 43 33 | 0.91 |  |

**Supplementary Table 2- Comprehensive Thematic Table with Goleman EI Domains**

| **No.** | **Author(s)** | **Year** | **Theme(s)** | **Linked Goleman EI Domain(s)** |
| --- | --- | --- | --- | --- |
| 1 | Baseer et al. | 2024 | Reflective Practices and Writing | Empathy; Self-Awareness |
| 2 | Mahmoudi et al. | 2024 | Narrative and Storytelling Interventions | Empathy; Self-Awareness; Social Skills |
| 3 | Arumugam et al. | 2024 | Communication Skills Training | Effective Communication (Social Skills); Empathy |
| 4 | Kanagasabai et al. | 2024 | Narrative and Storytelling Interventions | Empathy; Self-Awareness; Social Skills |
| 5 | Rhines et al. | 2024 | Emotional Intelligence Enhancement | Self-Regulation; Motivation; Empathy |
| 6 | Kadam et al. | 2024 | Reflective Practices and Writing | Self-Awareness; Empathy |
| 7 | Khorasani et al. | 2023 | Emotional Intelligence Enhancement | Self-Regulation; Empathy; Motivation |
| 8 | Rezaei et al. | 2023 | Reflective Practices and Writing | Self-Awareness; Empathy |
| 9 | Kagawa et al. | 2023 | Narrative and Storytelling Interventions | Empathy; Social Skills |
| 10 | Yuen et al. | 2023 | Communication Skills Training | Effective Communication (Social Skills); Empathy |
| 11 | Tariq et al. | 2023 | Experiential Learning and Patient Exposure | Empathy; Self-Awareness; Social Skills |
| 12 | Lisevick et al. | 2023 | Emotional Intelligence Enhancement | Empathy; Self-Awareness; Social Skills; Motivation |
| 13 | Donisi et al. | 2022 | Communication Skills Training | Empathy; Self-Regulation; Social Skills |
| 14 | Bętkowska-Korpała et al. | 2022 | Personalized Interventions and Diversity Considerations | Self-Awareness; Empathy |
| 15 | Potts et al. | 2022 | Personalized Interventions and Diversity Considerations | Empathy; Social Skills; Self-Awareness |
| 16 | Ardenghi et al. | 2022 | Emotional Intelligence Enhancement | Self-Regulation; Empathy; Social Skills |
| 17 | G C, Krishna Bahadur et al. | 2022 | Narrative and Storytelling Interventions | Empathy; Self-Awareness; Social Skills |
| 18 | Lam et al. | 2022 | Experiential Learning and Patient Exposure / Stress, Burnout, and Coping Interventions | Empathy; Self-Regulation |
| 19 | Olsen & Gebremariam | 2022 | Not reported | *Not Reported* |
| 20 | Epinat-Duclos et al. | 2021 | Communication Skills Training | Empathy; Social Skills |
| 21 | Praharaj et al. | 2021 | Personalized Interventions and Diversity Considerations | Empathy; Self-Awareness |
| 22 | Fukuyasu et al. | 2021 | Emotional Intelligence Enhancement | Empathy; Self-Regulation; Social Skills |
| 23 | Przymuszała et al. | 2021 | Communication Skills Training | Social Skills; Motivation |
| 24 | Van Winkle et al. | 2021 | Communication Skills Training / Experiential Learning (Remote) | Empathy; Social Skills; Self-Awareness |
| 25 | Yang et al. | 2021 | Experiential Learning and Patient Exposure | Empathy; Self-Awareness; Social Skills |
| 26 | Airagnes et al. | 2021 | Emotional Intelligence Enhancement | Empathy; Self-Awareness |
| 27 | Imperato & Strano-Paul | 2021 | Reflective Practices and Writing | Empathy; Self-Awareness |
| 28 | Jacoby et al. | 2021 | Emotional Intelligence Enhancement / Stress, Burnout, and Coping Interventions | Empathy; Self-Regulation; Motivation |
| 29 | Britz et al. | 2024 | Assessment Tools and Structural Interventions | Empathy; Social Skills |
| 30 | Neeley et al. | 2024 | Narrative and Storytelling Interventions | Empathy; Self-Awareness |
| 31 | Hashim et al. | 2024 | Reflective Practices and Writing | Self-Awareness; Empathy |
| 32 | Pearlman Shapiro et al. | 2023 | Experiential Learning and Patient Exposure | Empathy; Social Skills; Self-Awareness |
| 33 | Ward & Howick | 2023 | Communication Skills Training | Social Skills; Empathy; Self-Regulation |
| 34 | Leijenaar et al. | 2023 | Narrative and Storytelling Interventions | Empathy; Self-Awareness; Social Skills |
| 35 | Erlich et al. | 2023 | Reflective Practices and Writing | Empathy; Self-Awareness |
| 36 | Nandagopal & Walker | 2022 | Reflective Practices and Writing | Empathy; Self-Awareness |
| 37 | Ng et al. | 2022 | Reflective Practices and Writing | Self-Awareness; Empathy |
| 38 | Bukowski et al. | 2022 | Communication skills training/Experiential Learning | Empathy, Social skills |
| 39 | Kikukawa et al. | 2021 | Experiential Learning and Patient Exposure | Empathy; Self-Awareness; Social Skills |
| 40 | D, Savitha et al. | 2021 | Reflective Practices and Writing | Empathy; Self-Awareness |
| 41 | Cecchetti et al. | 2021 | Communication Skills Training | Empathy; Self-Regulation |
| 42 | Rieffestahl et al. | 2021 | Experiential Learning and Patient Exposure | Empathy; Self-Awareness |
| 43 | Laughey et al. | 2021 | Reflective Practices and Writing | Empathy; Self-Awareness |
| 44 | Abrams et al. | 2021 | Experiential Learning and Patient Exposure / Reflective Practices and Writing | Empathy; Self-Awareness; Social Skills |
| 45 | Blalock et al. | 2024 | Assessment Tools and Structural Interventions | Empathy; Social Skills |
